# Outcome of *Pneumocystis Jirovecii* pneumonia (PcP) in post-CAR-T patients with hematological malignancies

**DOI:** 10.1186/s12879-024-09893-x

**Published:** 2024-10-13

**Authors:** Cheng Zu, Wenxiao Li, Mingming Zhang, Yetian Dong, Shan Fu, Jingjing Feng, Ruimin Hong, He Huang, Yongxian Hu, Junwei Su

**Affiliations:** 1 Bone Marrow Transplantation Center, the First Affiliated Hospital, Zhejiang University School of Medicine, No.79 Qingchun Road, Hangzhou, China; 2 Liangzhu Laboratory, Zhejiang University Medical Center, 1369 West Wenyi Road, Hangzhou, 311121 China; 3 Institute of Hematology, Zhejiang University, Hangzhou, China; 4 Zhejiang Province Engineering Laboratory for Stem Cell and Immunity Therapy, Hangzhou, China; 5 The Department of Infectious Diseases, State Key Laboratory for Diagnosis and Treatment of Infectious Diseases, National Clinical Research Center for Infectious Diseases, Collaborative Innovation Center for Diagnosis and Treatment of Infectious Diseases, the First Affiliated Hospital, School of Medicine, Zhejiang University, No. 79 Qingchun Road, Hangzhou, China

**Keywords:** Pneumocystis pneumonia, Hematological malignancy, Adoptive cell therapy, Primary prophylaxis

## Abstract

**Background:**

*Pneumocystis jirovecii* pneumonia (PcP) is an opportunistic infection associated with immunocompromised patients. The development of novel immunotherapies has promoted the incidence of PcP. This study describes the clinical course and outcome of PcP in chimeric antigen receptor (CAR) T cell recipients with hematological malignancies.

**Methods:**

This is a retrospective case series of CAR-T recipients diagnosed with PcP in our center. The cases were all confirmed by metagenomic next-generation sequencing of clinical samples. The demographic, clinical, and outcome data were retrieved from the patients’ medical charts and electronic medical record system.

**Results:**

In total, 8 cases of PcP were identified. The underlying malignancies included T-acute lymphoblastic leukemia (ALL) (*n* = 1), diffuse large B cell lymphoma (DLBCL) (*n* = 4), and B-ALL (*n* = 3). One patient received short-term sulfamethoxazole-trimethoprim (SMZ-TMP) while the others had no prophylaxis. Four patients had neutropenia/lymphopenia at the diagnosis of PcP, and two patients had immunosuppressants within one month before PcP manifestation. The median time from CAR-T infusion to PcP diagnosis was 98.5 days (range 52–251). Seven patients recovered from PcP after proper management while one died of septic shock.

**Conclusion:**

PcP can occur after different CAR-T product, and the long-term depletion of immune cells seems to be related to PcP. SMZ-TMP is effective in this setting. More real-world experience of CAR-T therapy is required to assess the incidence and outcome of PcP in this population.

**Supplementary Information:**

The online version contains supplementary material available at 10.1186/s12879-024-09893-x.

## Introduction

*Pneumocystis jirovecii* is an opportunistic pathogen which causes *Pneumocystis* pneumonia (PcP), a life-threatening infection mostly seen in human immunodeficiency virus (HIV)-infected patients. As novel immunotherapies are now used in a wide spectrum of diseases, non-HIV, immunocompromised population at risk of PcP are increasing because of iatrogenic immunosuppression [1]. The incidence, course, and outcome of PcP in the setting of rituximab therapy have been widely reported [2], while PcP cases in recipients of another immunotherapy with similar mechanism — chimeric antigen receptor (CAR)-T therapy — have been scarce. Despite the unprecedented efficacy in hematological malignancies, CAR-T cells induce profound immune cell depletion resulting in an immunocompromised state, to which the underlying malignancies and prior treatments also contribute [3].

Particularly, the most widely used CAR-T therapy against CD19^+^ cells could induce, along with the ongoing response, long-term B-cell aplasia and subsequent hypogammaglobulinemia, which would impair both humoral and cellular immunity [4]. Hill et al*.* have proposed three phases to depict the susceptibility to different opportunistic infections after CD19-targeted CAR-T cell therapy [5]. As they suggested, most post-CAR-T infections have been bacterial in the first 28 days after infusion, while viruses, especially intermittent respiratory viruses predominate in late time points. As a severe opportunistic infection complication, *Pneumocystis jirovecii* pneumonia (PJP) have been rare and tend to occur in late phases [6, 7]. Given the lack of long-term follow-up in CAR-T trials, in addition to the scarcity of this disease, the harm of PJP has been long underestimated in CAR-T recipients, and the necessity of active prophylaxis against PJP has also been long debated on.

In this retrospective report, we present 8 cases of PJP post different CAR-T therapies, in order to emphasize the need for prophylaxis, early diagnosis, treatment and outcome of PJP in CAR-T recipients.

## Methods

CAR-T recipeints diagnosed with PcP within follow-up at our center from November 2020 to September 2022 were included.

A diagnosis of PcP was established when all three of the following criteria were met: 1) *P. jirovecii* DNA detected by metagenomic next-generation sequencing (mNGS); 2) characteristic radiological manifestations in chest computed tomography (CT), mostly featured by disseminated ground-glass pattern; 3) clinical symptoms of pneumonia.

Neutropenia was defined as a neutrophil count < 1000/mL, while lymphopenia was defined as a lymphocyte count < 500/mL.

The clinical data were collected from the electronic medical record system.

## Results

### Cohort characteristics

During the specified time period, near 300 patients received CAR-T therapy in our center, among whom 8 patients were diagnosed with PcP during long-term follow-up. The median age was 60 years-old (range 50–70) at PcP diagnosis. One patient had a history of allogeneic hematopoietic stem cell transplantation. The underlying malignancies included T-acute lymphoblastic leukemia (ALL) (*n* = 1), diffuse large B cell lymphoma (DLBCL) (*n* = 4), and B-ALL (*n* = 3), and in accordance with it, CAR-T cells targeting CD7, CD19, and CD22 were infused, respectively. All patients with leukemia reached complete remission/response (CR), 1 with positive minimal residual disease (MRD) and 3 with negative MRD; while 3 patients with DLBCL achieved CR and 1 partial response (PR). (Table 1).

**Table 1:** Baseline characteristics of patients

Patient No.	Gender	Age^a^	Primary Disease	Previous Cellular Therapy	CAR-T Target	Source of CAR-T	Lymphodepletion Regimen^b^	Best Response	Consolidation Therapies between Response and PcP	Immunosuppressive Agent(s) within 1 month before PcP	Prophylaxis for PcP after CAR-T Infusion	Last Neutropenia before PcP Manifestation (days)	Last Lymphopenia before PcP Manifestation (days)
1	Male	65	T-ALL	allo-HSCT	CD7	HSC Donor	FC	BM: CR, MRD(-) EMD: SD	None	None	None	Never	34
2	Female	65	DLBCL	None	CD19	Autologous	FACE	CR	PD-1 mAb	Steroid	None	5	72
3	Male	59	DLBCL	None	CD19	Autologous	FACE	CR	PD-1 mAb	None	None	88	94
4	Male	70	DLBCL	None	CD19	Autologous	FC	CR	None	None	None	0	0
5	Male	40	DLBCL	None	CD19	Autologous	FACE	PR	PD-1 mAb	None	SMZ-TMP	3	107
6	Female	61	B-ALL	CD19 CAR-T CD22 CAR-T	CD19-22	Autologous	FC	CR, MRD(-)	None	None	None	0	0
7	Female	50	B-ALL	CD19 CAR-T CD22 CAR-T CD19 + CD22 CAR-T	CD19	Autologous	FCE	CR, MRD(-)	None	None	None	24	0
8	Male	53	B-ALL	CD19 CAR-T	CD19	Autologous	FC	CR, MRD( +)	Multiple Chemotherapy	1. Cytotoxic Chemotherapy 2. Steroid	None	0	0

*Abbreviations*: *ALL* acute lymphoid leukemia, *DLBCL* diffuse large B cell lymphoma, *allo* allogeneic, *HSCT* hematopoietic stem cell transplantation, *CAR-T* chimeric antigen receptor T cell, *HSC* hematopoietic stem cell, *BM* bone marrow, *CR* complete remission/response, *MRD* minimal residual disease, *EMD* extramedullary disease, *SD* stable disease, *PR* partial response, *PcP Pneumocystis jirovecii* pneumonia, *PD-1* programmed death-1, *mAb* monoclonal antibody, *SMZ-TMP* sulfamethoxazole-trimethoprim

^a^Age at CAR-T infusion

^b^F stands for fludarabine, C stands for cyclophosphamide, A stands for anthracycline, E stands for etoposide

### Immunosuppression and prophylaxis before PcP

**Fig. 1:**
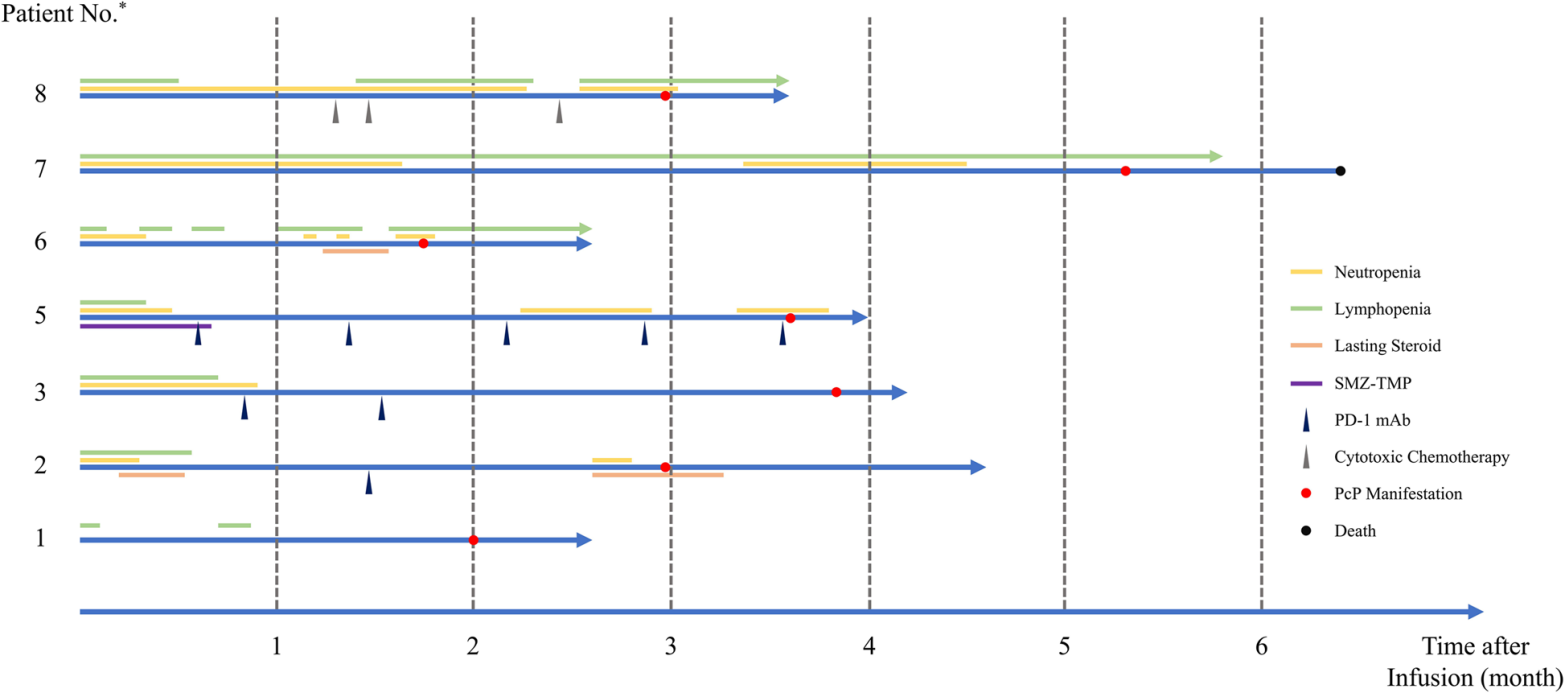
Brief timelines for each patients. Abbreviations: No., number; SMZ-TMP, sulfamethoxazole-trimethoprim; PD-1, programmed death-1; mAb, monoclonal antibody; PcP, *Pneumocystis jirovecii* pneumonia. ^*^Patient 4 was not included because of the incompleteness of his follow-up data

Two patients received immunosuppressive agents within 1 month before PcP manifestations, including corticosteroids (Patient 2, prednisone 30 mg daily; and Patient 8, dexamethasone 10 mg daily) and cytotoxic chemotherapies (Patient 8, because of the relapsed primary disease). Other treatments between CAR-T infusion and PcP manifestation which were assumed to be of no significant immunosuppression included sintilimab (Patients 2, 3, and 5 as consolidation for primary disease) and corticosteroids (Patient 4, methylprednisolone 4 mg daily). (Fig. 1).

Based on the protocols in our center, there has been no routine prophylaxis against *P. jirovecii* after infusion, while Patient 5 had been taking sulfamethoxazole-trimethoprim (SMZ-TMP) until 20 days after infusion because of suspected PcP during the myelosuppression resulted from previous chemotherapy.

### Clinical course and outcome

Brief timelines for each patients are presented in Fig. 1. Leukopenia was transient in Patients 1, 2, and 3, while Patients 5, 6, 7, and 8 developed PcP during recurrent or persistent neutropenia and/or lymphopenia.

All the cases of pneumonia manifested at least 50 days from CAR-T infusion (52–251 days, median 98.5 days). The clinical presentations and outcome are summarized in Table 2. Key laboratory examinations at diagnosis are presented in Supplementary Table 1. Most patients presented mild manifestations consisting of fever and nonproductive cough, with CT images showing diffused ground-glass patterns (Supplementary Fig. 1), and recovered after treatment. The anti-infection regimens were based on SMZ-TMP and caspofungin, steroids and immunoglobulin were used for excessive inflammation if necessary (for detailed anti-infection regimen, please refer to Supplementary Table 2). Three patients had comorbid viral and bacterial infections, including 2 with tuberculosis. Fortunately, 7 out of 8 patients recovered from PcP after treatment, while one (Patient 4) died of septic shock resulted from systemic infection. Given the emergency, he was treated at a lower local hospital rather than at our center, and detailed disease course was not available.

**Table 2:** PcP-related manifestations, treatments, and outcomes

Patient No.	Time from CAR-T Infusion to PcP Manifestation (days)	Initial Manifestations	Primary Disease Status at PcP diagnosis	Decisive Diagnosing Technique (Copy Number)	PcP Treatment	PcP outcome	Infectious Co-morbidity (Pathogen/Site)
1	60	1. Fever 2. Cough	BM: CR, MRD(-) EMD: SD	mNGS of BALF (3066)	1. SMZ-TMP 2. Caspofungin 3. Steroid	Remission	None
2	89	1. Fever 2. Cough	CR	mNGS of BALF (8)	1. SMZ-TMP 2. Caspofungin 3. Steroid 4. IVIg	Remission	None
3	115	1. Fever 2. Cough	CR	mNGS of BALF (306)	1. SMZ-TMP 2. Caspofungin 3. Steroid	Remission	None
4	251	1. Fever	CR	mNGS of PB (^a^)	1. Caspofungin 2. Steroid	Death	None
5	108	1. Fever 2. Cough	PR	mNGS of BALF (5200)	1. SMZ-TMP 2. Caspofungin	Remission	1. *Mycobacterium tuberculosis*/BALF 2. CMV/Blood & Aqueous humour
6	52	1. Fever 2. Fatigue 3. Dyspnea	CR, MRD(-)	mNGS of PB (297)	1. SMZ-TMP 2. Caspofungin 3. Steroid	Remission	1. CMV/Blood 2. BKV/Blood
7	159	1. Fever 2. Cough 3. Dyspnea	CR, MRD(-)	mNGS of BALF (93825)	1. SMZ-TMP 2. Steroid	Remission	1. *Stenotrophomonas maltophilia*/BALF 2. *Mycobacterium tuberculosis*/BALF 3. Human Coronavirus NL63/BALF 4. CMV/Blood 5. CNS infection suspected without direct evidence
8	89	1. Fever 2. Cough	Relapsed	mNGS of BALF (1461)	1. SMZ-TMP 2. Caspofungin 3. Steroid	Remission	None

*Abbreviations*: *CAR-T* chimeric antigen receptor T cell, *PcP Pneumocystis jirovecii* pneumonia, *BM* bone marrow, *CR* complete remission/response, *MRD* minimal residual disease, *EMD* extramedullary disease, *SD* stable disease, *PR* partial response, *mNGS* metagenomic next-generation sequencing, *BALF* bronchoalveolar lavage fluid, *PB* peripheral blood, *SMZ-TMP* sulfamethoxazole-trimethoprim, *IVIg* intravenous immunoglobulin, *CMV* cytomegalovirus, *BKV* BK polyomavirus, *CNS* central nervous system

^a^Detailed mNGS report is not available because Patient 4 was treated in a local hospital and had no chance to transfer to our center

### Representative case

Patient 7 had struggled through multiple infections ever since CAR-T infusion, in concordance with the prolonged and profound lymphopenia and neutropenia. Half a year after CAR-T infusion, she presented at our center with dyspnea, productive cough, and fever. A chest CT suggested severe pneumonia (Supplementary Fig. 1), thus, a bronchoscopy was warranted to get quick and accurate identification of possible pathogens. mNGS of BALF detected various pathogens including *P. jiroveci, Stenotrophomonas maltophilia*, *Mycobacterium tuberculosis*, and Human Coronavirus NL63. Meanwhile, cytomegalovirus reactivation was detected in peripheral blood. After the administration of combined drug treatment (meropenem for *S. maltophilia*, SMZ-TMP + caspofungin for *P. jiroveci*, ganciclovir for cytomegalovirus, isoniazid + ethambutol for tuberculosis, and linezolid to prevent potential additional infections), complex infection was mostly contained. Unfortunately, her impaired respiratory and immune systems were too vulnerable to resist another attack of severe acute respiratory syndrome coronavirus 2 (SARS-CoV2), and eventually died of consequent respiratory failure 1 month post the diagnosis of PcP.

## Discussion

PcP has long been recognized as an acquired immunodeficiency syndrome (AIDS)-defining illness, and one of the most common opportunistic infection in human immunodeficiency virus (HIV)-infected patients [8]. However, different from decades ago, more cases of PcP are currently found in non-HIV patients than in patients with it [9]. Common risk factors of PcP development in patients without HIV include the use of immunosuppressive agents, hematopoietic stem cell or solid organ transplantation, and malignancies or inflammatory conditions (particularly when cytotoxic or immunologic treatments are involved) [9, 10]. Although the use of CAR-T cells for treating various tumors is increasing, data concerning the incidence, outcome, and disease course of PcP in post-CAR-T setting is scarce. A single-center retrospective cohort study including 280 CD19 CAR-T recipients with B cell lymphoma reported a cumulative incidence of 1.07% (3/280) with prophylaxis in a median follow-up of 259 days; other studies with smaller sample sizes identified cumulative incidences range from 1.67% to 7.32% [6, 7, 11].

In this study, we described 8 cases of PcP in differently targeted CAR-T recipients, which shows that this complication is not restricted to CD19 CAR-T. Nonetheless, there is no established guideline or consensus on the prophylaxis in CAR-T recipients, and data currently available are far from enough to conduct evident-based conclusion. Considering the prevalence of *P. jirovecii* exposure/colonization in vast population [12, 13], the disease should be taken seriously in the real-world application of CAR-T cells. The experiences in hematopoietic stem cell transplantation recipients and rituximab recipients might be helpful. Routine prophylaxis against *P. jirovecii* is now widely accepted in these two patient populations, while the duration varies greatly from center to center [14, 15]. Even with prophylaxis, PcP cases have occasionally occurred right after the cessation, which shows that a defined period might not be optimal for every patient, personalized prophylactic regimen should be tailored.

To our knowledge, this is the largest case series describing PcP in post-CAR-T patients, and the first report of PcP in patients receiving CD7 CAR-T. The benefit of bacterial and viral prophylaxis after CAR-T therapy has been recognized by different recommendations [16, 17], while routine PcP prophylaxis is still under debate because of the rarity of the disease and possible severe adverse events, e.g., myelosuppression, induced by prophylactic agents. Although our case series does not establish a conclusion that CAR-T recipients must be given PcP prophylaxis, it does highlight the importance of being vigilant for PcP in such patients. Long-term follow-up of CAR-T recipients in the real-world setting will aid our understanding of immune reconstitution of CAR-T therapy and allow for personalized anti-PcP prophylaxis.

## Supplementary Information

Supplementary Material 1: Supplementary Table 1. Key laboratory examinations at diagnosis.

Supplementary Material 2: Supplementary Fig. 1. Representative CT images.

Supplementary Material 3: Supplementary Table 2. Detailed Anti-infection Regimens.

## Data Availability

The data presented in this study are available on request from the corresponding authors. The data is not publicly available due to the privacy of the patients.

## Abbreviations

PcP: *Pneumocystis **jirovecii* Pneumonia
CAR: Chimeric antigen receptor
ALL: Lymphoblastic leukemia
SMZ-TMP: Sulfamethoxazole-trimethoprim
HIV: Human immunodeficiency virus
mNGS: Metagenomic next-generation sequencing
CT: Computed tomography
DLBCL: Diffuse large B cell lymphoma
CR: Complete remission/response
MRD: Minimal residual disease
PR: Partial response
allo: Allogeneic
HSCT: Hematopoietic stem cell transplantation
HSC: Hematopoietic stem cell
BM: Bone marrow
EMD: Extramedullary disease
SD: Stable disease
PD-1: Programmed death-1
mAb: Monoclonal antibody
SARS-CoV2: Severe acute respiratory syndrome coronavirus 2
AIDS: Acquired immunodeficiency syndrome
