# Supplementary material for: Outcome of Pneumocystis Jirovecii pneumonia (PcP) in post-CAR-T patients with hematological malignancies
Source: BMC Infect Dis. 2024 Oct 13;24:1147. doi: 10.1186/s12879-024-09893-x (PMC11472446; doi:10.1186/s12879-024-09893-x)
Supplement: Supplementary file 1 — Supplementary Material 1: Supplementary Table 1. Key laboratory examinations at diagnosis. [file 12879_2024_9893_MOESM1_ESM.docx]

**Supplementary Table 1. Key laboratory examinations at diagnosis.**

| Patient | LDH (U/L) | β-glucan (pg/ml) | CD4+ T (×109/L) |
| --- | --- | --- | --- |
| 1 | 268 | NA | 0.10 |
| 2 | 299 | NA | NA |
| 3 | 346 | NA | 0.19 |
| 4 | NA | NA | NA |
| 5 | 526 | NA | 0.27 |
| 6 | 623 | 235.2 | 0.11 |
| 7 | 606 | <10 | NA |
| 8 | 313 | <10 | 0.01 |
