# Supplementary figures and images for: Outcome of Pneumocystis Jirovecii pneumonia (PcP) in post-CAR-T patients with hematological malignancies
Source: BMC Infect Dis. 2024 Oct 13;24:1147. doi: 10.1186/s12879-024-09893-x (PMC11472446; doi:10.1186/s12879-024-09893-x)

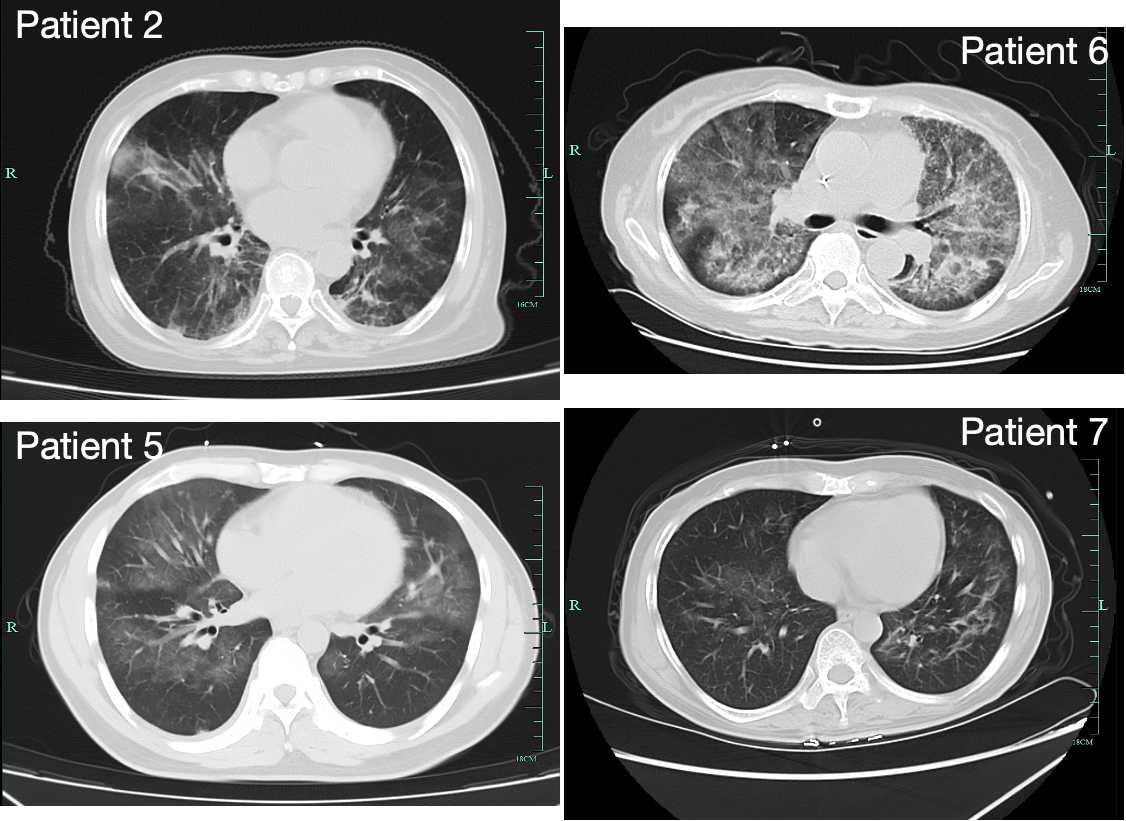


**Supplementary Figure 1. Representative CT images.**

Supplement: Supplementary file 2 — Supplementary Material 2: Supplementary Fig. 1. Representative CT images. [file 12879_2024_9893_MOESM2_ESM.docx]
