# Supplementary material for: Outcome of Pneumocystis Jirovecii pneumonia (PcP) in post-CAR-T patients with hematological malignancies
Source: BMC Infect Dis. 2024 Oct 13;24:1147. doi: 10.1186/s12879-024-09893-x (PMC11472446; doi:10.1186/s12879-024-09893-x)
Supplement: Supplementary file 3 — Supplementary Material 3: Supplementary Table 2. Detailed Anti-infection Regimens. [file 12879_2024_9893_MOESM3_ESM.docx]

**Supplementary Table 2. Detailed Anti-infection Regimens**

| Patient | SMZ-TMP | Other anti-infective treatment | Steroids |
| --- | --- | --- | --- |
| 1 | Sulfamethoxazole 800mg + Trimethoprim 160mg QID for 1 month | Caspofungin 50mg QD for 8 days | Methylprednisolone 40mg Q8h for 8 days (followed by oral tapering) |
| 2 | Sulfamethoxazole 800mg + Trimethoprim 160mg QID for 1 month | Caspofungin 50mg QD for 9 days Cefoperazone & Sulbactam 3g BID for 10 days Acyclovir 250mg Q8h for 9 days | Methylprednisolone 40mg Q8h for 12 days (followed by oral tapering) |
| 3 | Sulfamethoxazole 800mg + Trimethoprim 160mg TID for 1 month | Caspofungin 50mg QD for 7 days | Methylprednisolone 40mg Q8h for 9 days (followed by oral tapering) |
| 4 | NA^*^ | NA | NA |
| 5 | Sulfamethoxazole 800mg + Trimethoprim 160mg TID for 1 month | Caspofungin 50mg QD for 3 days | None |
| 6 | Sulfamethoxazole 1200mg + Trimethoprim 240mg TID or Sulfamethoxazole 800mg + Trimethoprim 160mg TID for 6 month (intermittently) | Caspofungin 50mg QD for 1 month | Methylprednisolone 40mg Q8h for 1 week Methylprednisolone 20mg Q8h for 1 week (followed by oral tapering) |
| 7 | Sulfamethoxazole 800mg + Trimethoprim 160mg QID for 3 weeks (until death) | Meropenem 1g Q8h for 1 week Moxifloxacin 0.4g QD for 1 week Linezolid 0.2g Q12h for 1 week | Methylprednisolone 40mg Q8h for 7 days (until death) |
| 8 | Sulfamethoxazole 1600mg + Trimethoprim 320mg TID for 1 month | Caspofungin 50mg QD for 15 days | Methylprednisolone 40mg Q8h for 10 days (followed by oral tapering) |

because he was treated in a local hospital and had no chance to transfer to our center.
